# Epidermodysplasia verruciformis–associated eccrine neoplasm: morphologic and immunohistochemical characterization of 25 lesions in two patients

**DOI:** 10.1007/s00428-026-04511-4

**Published:** 2026-04-17

**Authors:** Eric Araújo Lucas de Barros, Robert Lourenço Stoque Dias, Juliana de Sá Pires Carvalho, Isabelly Cristina Honorato de Queiroz, Carla Riama Lopes de Pádua Moura, Rafael de Deus Moura

**Affiliations:** 1 School of Medicine, Universidade Federal Do Piauí (UFPI), Teresina, Piauí Brazil; 2 Hospital Universitário, Universidade Federal Do Piauí (HU-UFPI), Teresina, Piauí Brazil; 3 Department of Specialized Medicine, Universidade Federal Do Piauí (UFPI), Teresina, Piauí Brazil

**Keywords:** Adnexal tumor, Atypia, Eccrine neoplasm, Epidermodysplasia verruciformis, Immunohistochemistry

## Abstract

We analyzed 25 eccrine neoplasms from two patients with hereditary epidermodysplasia verruciformis (EDV), representing the largest series reported to date. All lesions shared a reproducible architecture with multifocal epidermal connections, anastomosing epithelial strands of basaloid–poroid cells, and a myxoid fibrovascular stroma. Additional findings included focal clear cell change and intraepithelial atypia (20% of cases). Immunohistochemically, EMA and CEA confirmed ductal differentiation, while preserved YAP1 and negative NUT staining supported a lineage distinct from conventional poroma. p53 overexpression and diffuse p16 labeling were confined to atypical foci. These findings broaden the morphologic spectrum of this emerging entity and support the use of targeted immunohistochemistry to recognize atypical foci in EDV-associated eccrine neoplasms.

## Introduction

Patients with epidermodysplasia verruciformis (EDV) exhibit a marked predisposition to the development of non-melanoma skin cancers, reflecting the combined effects of β-HPV infection, ultraviolet radiation, and host genetic susceptibility [1]. Cutaneous squamous cell carcinoma is the most common malignancy, typically arising on chronically sun-exposed sites such as the face, neck, and upper limbs [1, 2]. These carcinomas often occur at a younger age in EDV and may behave more aggressively than sporadic counterparts. Basal cell carcinoma, although less frequent, has also been reported, supporting an expanded oncogenic spectrum in the setting of persistent β-HPV infection [3].

Recently, a distinct entity designated as EDV-associated eccrine neoplasm has been proposed to encompass a subset of adnexal tumors arising in the context of EDV [4]. To date, only one study has formally delineated this lesion as a separate histopathologic entity [4], whereas two additional reports have described morphologically similar eccrine or mixed adnexal neoplasms that likely fall within the same spectrum [5, 6]. To further delineate this emerging entity, we report 25 eccrine neoplasms from two patients with EDV and detail their clinicopathologic and immunohistochemical features, including a subset with intraepithelial atypia.

## Materials and methods

Two female patients with hereditary epidermodysplasia verruciformis (EDV) were identified in routine practice at two independent tertiary care centers. All available skin biopsy specimens from these patients were retrieved and reviewed for histopathologic re-evaluation. Tissue samples had been fixed in 10% neutral buffered formalin, paraffin-embedded, and routinely stained with hematoxylin and eosin (H&E).

Immunohistochemistry was performed on 4-µm formalin-fixed, paraffin-embedded sections using a Ventana BenchMark GX automated stainer (Ventana Medical Systems, Tucson, AZ) according to the manufacturer’s protocol. The antibody panel included EMA (E29, 1:200, Ventana), CEA (CEA31, ready-to-use, Ventana), p63 (4A4, ready-to-use, Ventana), p16 (E6H4, ready-to-use, Ventana), p53 (DO-7, 1:100, Ventana), YAP1 (63.7, 1:100, Cell Signaling Technology, Danvers, MA), and NUT (C52B1, 1:100, Cell Signaling Technology). Slides were counterstained with hematoxylin and evaluated by light microscopy.

## Results

**Fig. 1:**
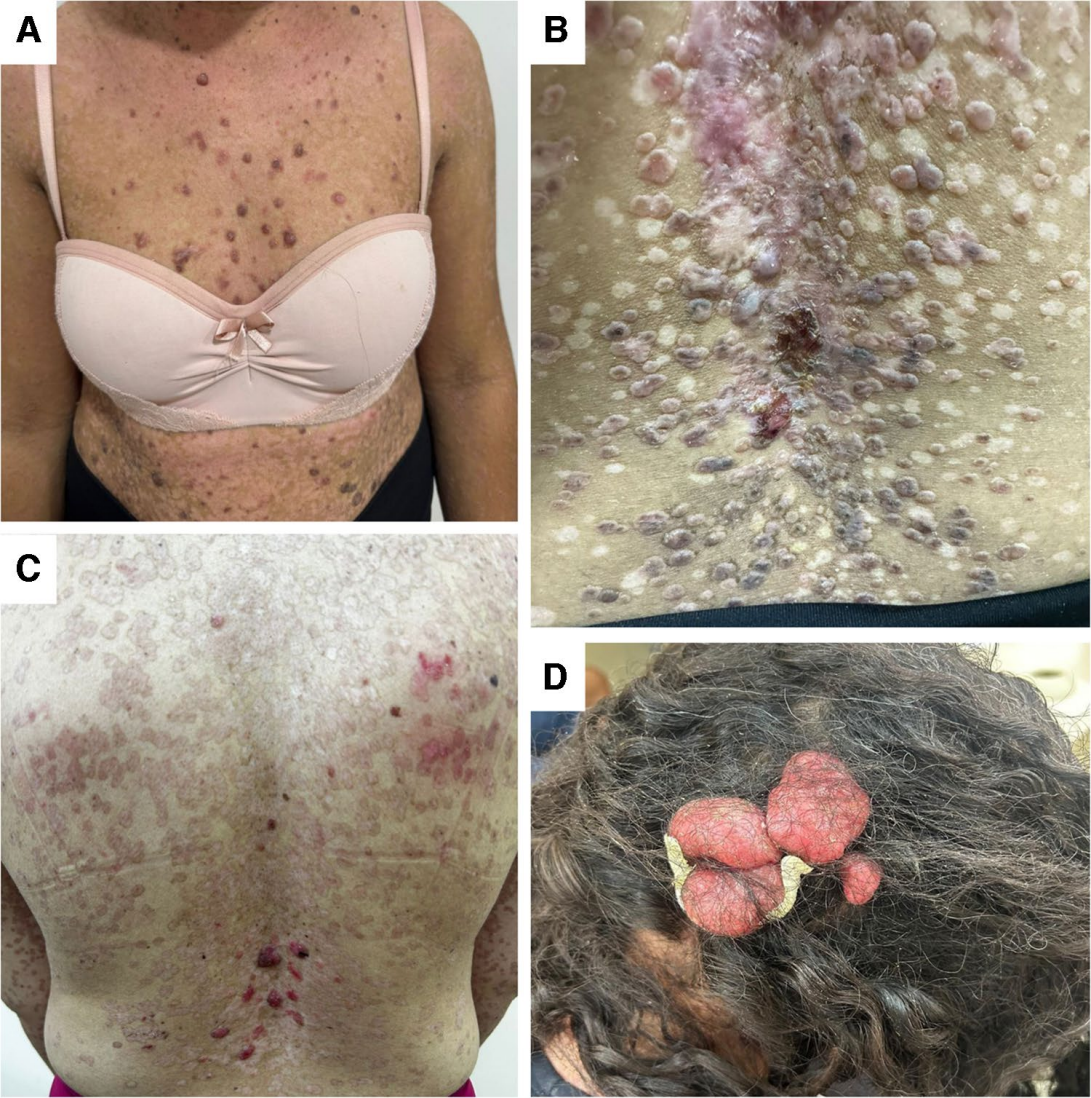
Patient 1 (**A**, **B**). **A** Erythematous-to-brownish papules and nodules on the anterior chest. **B** Pityriasis versicolor–like hypopigmented lesions and friable erythematous nodules on the vertebral region. Patient 2 (**C**, **D**). **C** Erythematous macules over the posterior trunk, along with several erythematous papules and nodules in the sacral region. **D** Three grouped nodules in the occipital region

Twenty-five cutaneous lesions from two female patients with hereditary epidermodysplasia verruciformis (EDV) were evaluated. Patient 1 (28 years) presented with erythematous-to-brownish papules and nodules on the anterior chest and abdomen, together with widespread hypopigmented macules and pityriasis versicolor–like changes on the trunk and limbs, and hyperpigmented papules coalescing into plaques with friable erythematous nodules on the vertebral region (Fig. 1A, B). Patient 2 (40 years) showed widely distributed hyperpigmented, mildly erythematous macules on the posterior trunk, erythematous papules and nodules in the sacral region, and grouped occipital nodules (Fig. 1C, D). Most lesions were sampled from the back and shoulder (median size 6 mm; range 0.7–45 mm). Clinically, the leading impression was cutaneous squamous cell carcinoma, and several eccrine neoplasms were incidental microscopic findings in skin adjacent to other clinically evident tumors.

### Histopathological findings

**Fig. 2:**
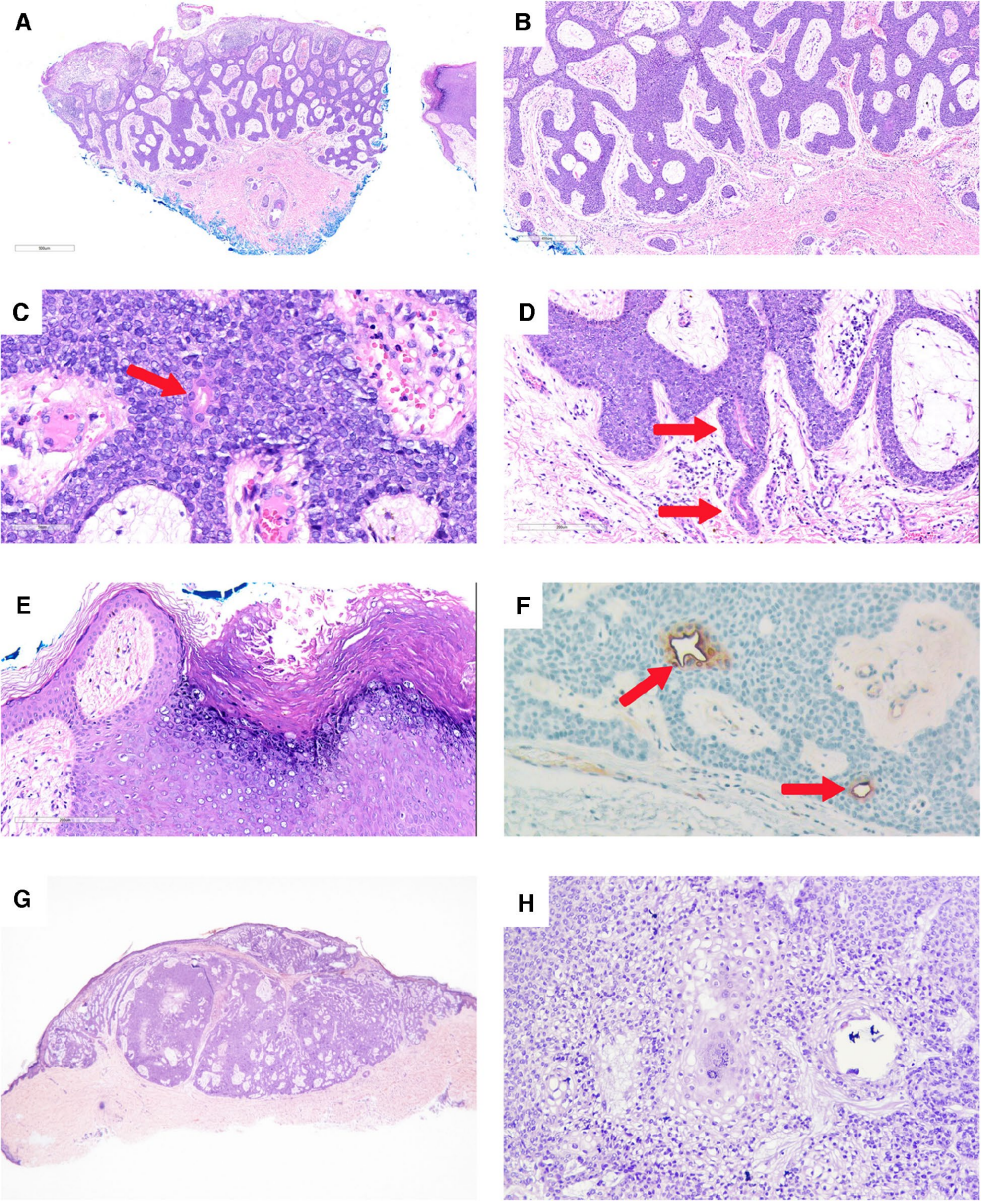
Histopathologic features of epidermodysplasia verruciformis–associated eccrine neoplasms. **A** Neoplasm demonstrating multifocal connections to the overlying epidermis (H&E, × 23). **B** Typical anastomosing epithelial pattern within a myxoid fibrovascular stroma. (H&E, × 53). **C** High-power view demonstrating the biphasic epithelial composition: a predominant population of basaloid-poroid cells and a minor population of cuticular cells lining ductal structures (arrow; H&E, × 400). **D** Tumor tracking along a pre-existing eccrine duct (arrows; H&E, × 200). **E** Cytopathic changes of epidermodysplasia verruciformis (EDV) in the adjacent epidermis, characterized by keratinocytes with pale grayish-blue cytoplasm (H&E, × 113). **F** CEA immunohistochemistry highlighting luminal ductal differentiation within the neoplastic epithelium (arrow; × 200). **G** EDV-associated eccrine neoplasm with clear cell change. Low-power view demonstrating multifocal epidermal connections and an anastomosing epithelial architecture (H&E, × 20). **H** Medium-power view highlighting clear-cell change within the neoplastic epithelium (H&E, × 100)

Histopathologically, all lesions shared a reproducible pattern characterized by multifocal connections to the overlying epidermis and a myxoid fibrovascular stroma (Fig. 2A, B). The epithelial component consisted predominantly of basaloid–poroid cells with scant cytoplasm and oval hyperchromatic nuclei, admixed with a smaller population of cuticular cells lining duct-like lumina (Fig. 2C). In focal areas, the proliferation tracked along a pre-existing eccrine duct (Fig. 2D), and the duct-like lumina showed architectural continuity with the tumor, supporting true neoplastic ductal differentiation rather than entrapped ducts. A lymphoplasmacytic stromal infiltrate was frequent, follicular cysts were common, and ulceration was present in approximately half of the lesions. Cytopathic changes of EDV were consistently present in the epidermis adjacent to the tumors (Fig. 2E). Clear cell change within the neoplastic epithelium was identified in three lesions (Fig. 2G, H).

**Fig. 3:**
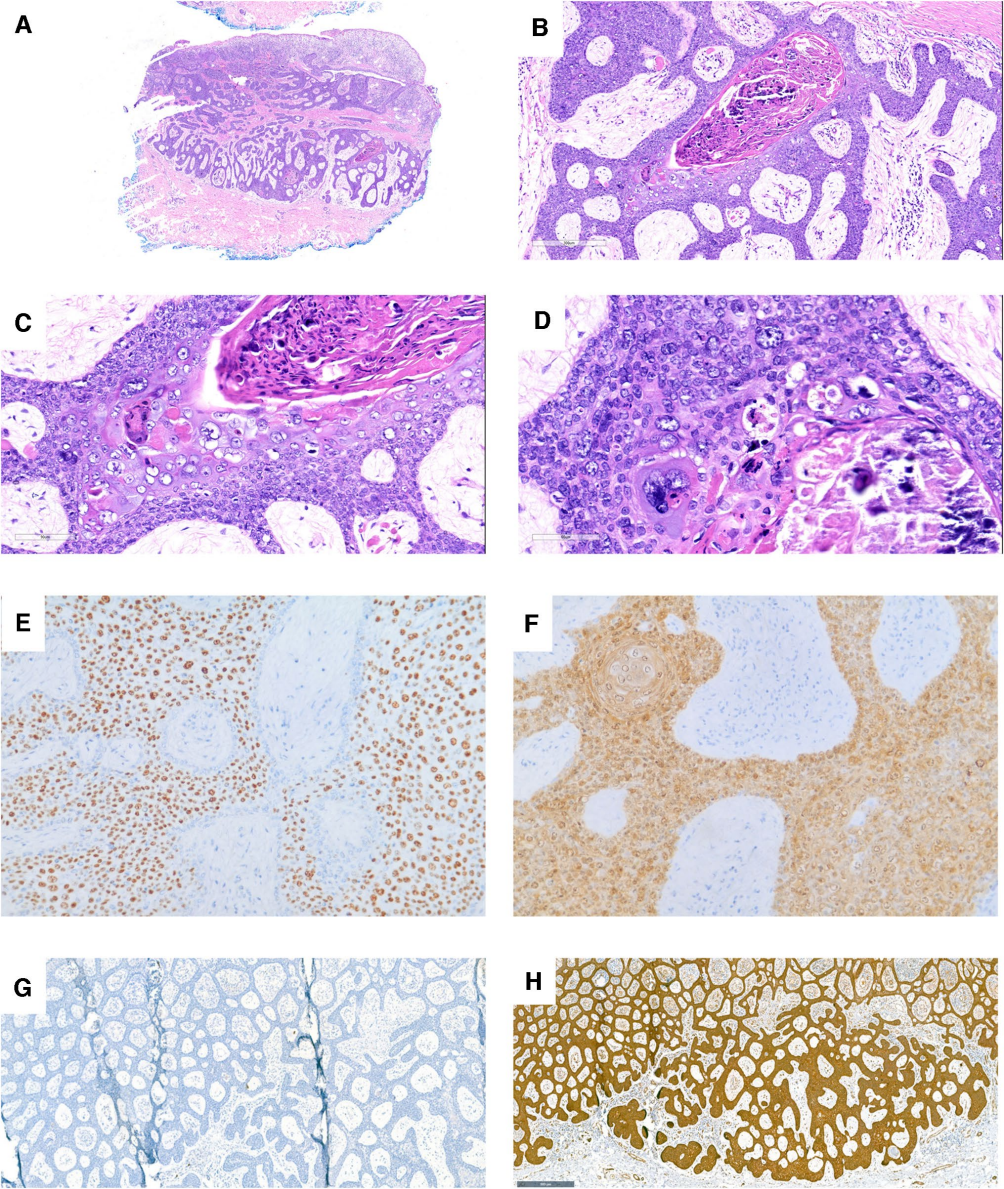
Intraepithelial atypia in epidermodysplasia verruciformis–associated eccrine neoplasm. **A** Low-power view of ulcerated lesion showing the typical architecture of the eccrine neoplasm with connections to the epidermis (H&E, × 14). **B**, **C** Medium magnification illustrating areas of mass necrosis and prominent nuclear pleomorphism and atypia (H&E, × 87 and × 190, respectively). **D** High-power magnification detailing intense nuclear pleomorphism (H&E, × 335). **E** Immunohistochemistry showing strong diffuse p53 overexpression in neoplastic cells (× 200). **F** Diffuse p16 positivity in neoplastic cells within the atypical focus (× 200). **G** Absence of NUT expression (× 20). **H** Preserved nuclear YAP1 expression (× 20)

Foci meeting criteria for intraepithelial atypia (pleomorphism, mitotic activity and/or necrosis en masse, without stromal invasion) were identified in 5 lesions (Fig. 3A–D). Other tumors diagnosed in these two patients during the same period included invasive squamous cell carcinoma, squamous cell carcinoma in situ, actinic keratosis, seborrheic keratosis, and plane wart.

On initial sign-out, most lesions were recognized as EDV-associated eccrine neoplasm, but a substantial subset required revision on re-evaluation, most commonly having been misclassified as poroma, basal cell carcinoma, or hidradenoma. On final review, 20 lesions were classified as EDV-associated eccrine neoplasm and 5 as EDV-associated eccrine neoplasm with intraepithelial atypia.

Immunohistochemically, EMA and CEA consistently highlighted ductal eccrine differentiation in all tested tumors (12/12 and 11/11, respectively) (Fig. 2F). In lesions with intraepithelial atypia, p53 overexpression and diffuse p16 labeling were common (4/4 and 3/4 tested cases, respectively) and were confined to the atypical foci (Fig. 3E, F). For differential diagnostic purposes, particularly to distinguish from poroma, all tested tumors were NUT-negative (10/10) and retained nuclear YAP1 expression (19/19) (Fig. 3G, H).

## Discussion

This study expands the clinicopathologic and immunohistochemical characterization of EDV-associated eccrine neoplasm, an emerging adnexal entity that remains sparsely documented. By reviewing 25 lesions from two patients with hereditary EDV and correlating clinical data, histopathology, and a focused immunohistochemical panel, we identified a reproducible diagnostic pattern. The core architectural features—multifocal epidermal connections, an anastomosing epithelial growth pattern, and a myxoid fibrovascular stroma—were present in all lesions. Basaloid–poroid cytomorphology with consistent ductal differentiation was also uniform. Frequent accompanying findings included a lymphoplasmacytic stromal infiltrate and follicular cyst formation, which may further support recognition of this lesion in routine practice.

To date, only a small number of publications have described EDV-associated eccrine neoplasms or closely related adnexal proliferations, together encompassing a total of 15 lesions [4–6]. By sharp contrast, our series includes 25 tumors, providing the most extensive dataset for comparative analysis to date. Importantly, the defining features emphasized in prior reports—epidermal connections, anastomosing strands/cords, poroid/basaloid cytomorphology, and demonstrable ductal differentiation—closely match the findings in our cohort, supporting diagnostic consistency across studies. Beyond confirming these criteria, we observed additional histopathologic variation, including focal clear-cell change within the neoplastic epithelium, which broadens the recognized morphologic spectrum.

Beyond morphology, our experience suggests that EDV-associated eccrine neoplasm may be under-recognized in EDV. In these two patients, it was identified more often than invasive squamous cell carcinoma, squamous cell carcinoma in situ, or actinic keratosis [1, 7]. Furthermore, review of the original pathology reports revealed that a substantial number of these lesions were initially misdiagnosed as other adnexal tumors, specifically poroma (4 cases), basal cell carcinoma (2 cases), and hidradenoma (1 case), which were subsequently reclassified as EDV-associated eccrine neoplasm. This pattern of diagnostic error emphasizes the need for increased awareness of this distinct entity.

Immunohistochemistry was particularly useful for confirming lineage and addressing key differentials. EMA and CEA were consistently expressed in patterns supporting ductal differentiation, corroborating prior observations [6]. Because conventional poromas and porocarcinomas commonly show YAP1-rearrangement–associated profiles (including loss of C-terminal YAP1 expression and/or aberrant NUT positivity in relevant fusion subsets), we applied YAP1 and NUT as adjunctive markers. Preserved YAP1 expression and negative NUT staining in tested cases argue against a conventional poroma spectrum lesion and support EDV-associated eccrine neoplasm as a biologically distinct process, in line with recent descriptions [4, 8].

Importantly, our study provides the first detailed characterization of the immunophenotypic changes associated with intraepithelial atypia in EDV-associated eccrine neoplasms. In our cohort, p53 overexpression and p16 positivity strongly correlated with cytologic atypia, being present in 100.0% and 75.0% of tested cases, respectively. Notably, these alterations were strictly confined to the neoplastic foci showing cytologic atypia, in sharp contrast to the benign background. This abrupt morphologic and immunophenotypic transition is most consistent with an early carcinoma in situ–equivalent change; however, in the EDV/β-HPV setting, it is not possible to exclude that at least some atypical changes may be influenced by HPV-related cytopathic effects and associated cell-cycle deregulation. Additionally, p16 expression was observed in benign cuticular cells lining ducts, mirroring the patchy, ductal-accentuated pattern described in poromas [9]. This represents a potential diagnostic pitfall and requires distinction from diffuse, strong labeling in pleomorphic atypical cells. Collectively, these findings suggest that—analogous to the distinction between poroma and porocarcinoma—p53 and p16 may serve as useful ancillary markers to support recognition of early intraepithelial transformation in EDV-associated eccrine neoplasms.

The main limitation of this study is that all lesions arose in only two patients with hereditary EDV, which restricts generalizability—particularly for conclusions regarding intraepithelial atypia. Future studies across a broader spectrum of EDV (including acquired forms) and with more systematic sampling will be required to define the full morphologic range and to clarify the biological significance of intraepithelial atypia.

In summary, this largest-to-date series defines a highly reproducible morphologic profile for EDV-associated eccrine neoplasm, expands its spectrum to include clear-cell change, and highlights common diagnostic pitfalls. EMA/CEA reliably document ductal differentiation, while YAP1/NUT profiles may help separate this entity from conventional poroma-spectrum tumors. Intraepithelial atypia can occur and warrants careful histologic assessment; when present, p53 and p16 may provide supportive evidence.

## Data Availability

The data supporting the findings of this study are derived from clinical and histopathologic records and are not publicly available due to ethical and privacy restrictions. De-identified data may be made available from the corresponding author upon reasonable request and subject to approval by the institutional Research Ethics Committee.

## Declarations

### Artificial intelligence statement

Artificial intelligence–based tools were used solely for language editing and grammatical revision. These tools did not influence data analysis, interpretation, or conclusions.
